# Patient safety culture among community pharmacists in Cairo

**DOI:** 10.1186/s42506-023-00136-6

**Published:** 2023-05-16

**Authors:** Mahi Al-Tehewy, Samera Mohamed, Noura Ammar

**Affiliations:** 1 Department of Community Medicine, Faculty of Medicine, Ain Shams University, Cairo, Egypt; 2 Memorial Ophthalmic Institute, Cairo, Egypt

**Keywords:** Patient safety, Safety culture, Community pharmacy

## Abstract

**Background:**

Measuring perception of patient safety culture among pharmacists working in community pharmacies is crucial to identify opportunities and areas that require improvement. The aim of this work is to measure patient safety culture among pharmacists working in community pharmacies in Cairo.

**Methods:**

A cross-sectional study was conducted among pharmacists working in community pharmacies in the center and south regions of Cairo. The Pharmacy Survey on Patient Safety Culture (PSOPSC), developed by the Agency for Healthcare Research and Quality (AHRQ) was used to collect data.

**Results:**

The study included 210 community pharmacies with a 95% response rate. The mean age of pharmacists was 28 ± 5.4 years. The overall positive response percentage (PRP) ranged between 35 and 69% with a mean of 57.4%. The highest PRP was identified in the domains of “teamwork” (68.97%), “organizational learning–continuous improvement” (64.93%) and “patient counseling” (61.83%). The PRP was less than 60% in 6 out of the 11 composites. The lowest PRP was found in the domain of “staffing, work pressure, and pace”, which scored 34.98%.

**Conclusion:**

The study identified areas of patient safety culture that require improvement in community pharmacies, especially in allocating staff, appropriate working hours, and training community pharmacists on the importance and principles of patient safety. The overall mean PRP of patient safety culture among community pharmacists highlights the need to include patient safety as the strategic priority at the level of community pharmacies.

## Introduction

Patient safety is a key pillar of healthcare quality. In recent years, there has been growing interest in patient safety movement. This stimulated research to measure and report on organizational attributes that are believed to promote patient safety. One such attribute is safety culture, defined as a product of individual and group values, attitudes, perceptions, competencies, and patterns of behavior that determine the commitment to, and the style and proficiency of an organization’s health and safety management [1].

Promoting safety culture in community pharmacies becomes a pressing issue as community pharmacies expand their roles from their traditional functions of efficiently dispensing prescriptions [2] to include other services such as immunization which is greatly obvious in supporting the delivery of the coronavirus (COVID-19) vaccination roll-out [3], delivering public health interventions as smoking cessation programs, weight management programs and others [4].

Many tools have been developed for evaluation of patient safety culture [5]. Measuring patient safety in community pharmacies can provide insights that can significantly contribute to organizational quality improvement efforts by raising staff awareness about patient safety as well as identifying areas of strengths and those that require improvement.

The Pharmacy Survey on Patient Safety Culture (PSOPSC) developed by AHRQ (The Agency for Healthcare Research and Quality) is designed specifically for community pharmacies. It measures safety dimensions as they relate to the work environment, communication among pharmacy staff, error mitigation, error documentation, and error handling as well as staff perception about the overall safety rating of the pharmacy [6].

Most of the research has always focused on patient safety culture in hospital settings. Little is known about patient safety practices and safety culture in community pharmacies. These care environments continue to be an essential but underappreciated part of the patient care system. The aim of this study was to measure patient safety culture dimensions using PSOPC and to identify factors affecting patient safety culture among pharmacists working in some community pharmacies in center and south of Cairo, Egypt.

## Methods

### Study design and setting

A cross-sectional study was conducted among a purposive sample of pharmacists who work in community pharmacies in central and southern Cairo.

### Study sample

A sample size of 210 pharmacists was calculated using Epi-info 7 software at a 95% confidence level and a margin of error of ± 0.05, based on the results of a previous study conducted by Alsaleh et al. 2018 in Kuwait [7] who found that the proportion of pharmacists with positive response for patient safety culture was 83.7%. Due to the unavailability of a list with the names and addresses of all community pharmacies in Cairo, a purposive sample of pharmacists who work in community pharmacies in central and southern Cairo was included in the study. The selection was based upon the accessibility of these regions to the investigators. If a pharmacy employed more than one pharmacist, only the most senior pharmacist who had worked for a long time in the pharmacy and was aware of all the details of the working place was invited to participate to prevent shared opinions.

Three hundred fifty-one community pharmacies were invited to complete the questionnaire; however, 70 pharmacies were operating without a pharmacy on duty during the visit time, and 61 pharmacies refused to participate. Finally, 220 questionnaires were distributed and 210 were completed and included in the study. The response rate was 95.5% among those who agreed to participate.

### Data collection methods

#### Survey instrument

The Community Pharmacy Survey on Patient Safety Culture (CPSPC) was developed by the Agency for Healthcare Research and Quality (AHRQ) and was first released in October 2012. The survey is designed to measure 11 dimensions of organizational patient safety culture using 36 items, six composites measured perception, and five measured practices. The survey uses 5-point agreement scales ranging from (“strongly disagree” to “strongly agree”) as well as frequency scales ranging from (“never” to “always”). The survey also includes one question that asks participants to rate their pharmacy’s patient safety on a scale from poor to excellent scale.

The CPSPC was chosen to evaluate safety culture because the instrument has been specifically designed for community pharmacy. The survey is a validated tool, and the reliability of its dimensions and their items have been proven and was previously tested for validity and reliability [8]. Furthermore, the use of a standardized tool facilitates comparisons across pharmacies and regions. The PSOPSC is a self- administered questionnaire which was distributed in its original English language and individual responses were kept anonymous. To increase the response rate, community pharmacists were contacted personally in their workplace and the objectives of the study were fully explained. The questionnaire was administered participants were given sufficient time to complete the questionnaire, which was then collected in person on the second day. Data were collected until the target sample size was attained.

### Statistical analysis

The Statistical Package for Social Science (SPSS) software, version 23 was used for analyzing the data. The frequency and percentage were used for describing qualitative variables, and comparison between groups was done using chi-square test and Fisher’s exact tests. The level of significance was chosen as a *p* value of 0.05.

During data analysis, the Likert scale categories were combined to simplify data presentation. For positively worded items, the percent positive was calculated by combining the percentage of “strongly agree” and “agree” responses, or “always” and “most of the time” responses depending on the response categories used for the item. For negatively worded items, the percent negative was calculated by combining the percentage of “strongly disagree” and “disagree” responses, or “never” and “rarely” responses.

## Results

A total of 210 community pharmacists from central and southern regions of Cairo participated in the study. Table 1 presents the demographic and professional characteristics of the participants. Pharmacists were predominantly males (54.8%), with mean age of 28.98(5.43), and years of experience of 6.05(5.18).

**Table 1:** Socio-demographic characteristics of the participating pharmacists working in community pharmacies in central and southern regions of Cairo in 2019–2020 (*n* = 210)

Variable	*n*	%
**Gender (*n* = 208)**		
Male	114	54.8
Female	94	45.2
**Age (*n* = 189)**		
Mean (SD)	28.98	(5.43)
**Years of experience in community pharmacy (*n* = 206)**		
Mean (SD)	6.05	(5.18)
**Position (*n* = 208)**		
Regular pharmacist	154	74.0
Manager	54	26.0
**Postgraduate studies (*n* = 207)**		
Yes	63	30.4
No	144	69.6

Tables 2 presents the responses of community pharmacy personnel regarding their perception of patient safety culture in their pharmacies. The dimensions of “Teamwork” and “Organizational Learning-Continuous Improvement” scored the highest positive response percentages (PRP). In particular, 75.4% of pharmacists had a positive perception of staff treating each other with respect in the “Teamwork” dimension, while 70.0% responded positively to the statement “When a mistake happens, we try to figure out what problems in the work process led to the mistake” in the “Organizational Learning-Continuous Improvement” dimension. Furthermore, 70.8% of pharmacists had a positive perception of the physical space and environment of their pharmacy being well-organized.

**Table 2:** The percentages of positive responses of the 11 patient safety culture composites of community pharmacy survey (*n* = 210)

Composite	Item	Positive response *N* (%)
**Perception of pharmacists about working in their community pharmacies**		
**Physical space and environment**	This pharmacy is well organized	148 (70.8%)
	This pharmacy is free of clutter	111 (53.1%)
	The physical layout support workflow	124 (59.6%)
**Teamwork**	Staff treat each other with respect	156 (75.4%)
	Staff clearly understand their roles and responsibilities	133 (63.9%)
	Staff work together as effective team	142 (67.6%)
**Staff training and Skills**	Technicians receive the training they need to do their jobs	115 (55.8%)
	Staff have the skills they need to do their jobs well	138 (67.6%)
	Staff who are new to this pharmacy receive adequate orientation	129 (62%)
	Staff get enough training from this pharmacy	125 (60%)
**Practices of community pharmacies on communication and work pace**		
**Communication openness**	Staff ideas and suggestions are valued	95 (45.7%)
	Staff feel comfortable asking questions when they are unsure about something	137 (65.9%)
	It is easy for staff to speak up to their supervisor about patient safety concerns	123 (59.1%)
**Patient counselling**	We encourage patients to talk to pharmacists about their medications	125 (60.7%)
	Our pharmacists spend enough time talking to patients about how to use their medications	126 (60%)
	Our pharmacists tell patients important information about their new prescriptions	136 (64.8%)
**Staffing work pressure and pace**	Staff take adequate breaks during their shifts	85 (41.0%)
	We feel rushed when processing prescriptions	78 (37.9%)
	We have enough staff to handle the workload	105 (50.25)
	Interruptions/distractions in this pharmacy make it difficult for staff to work accurately	80 (38.2%)
**Communication and prescription across shifts**	We have clear process about exchanging important prescription information across shifts	107 (53.2%)
	We have standard procedures for communicating prescription information across shifts	103 (49.8%)
	The status of problematic prescriptions is well communicated across shifts	97 (46.6%)
**Communication about mistakes**	Staff in this pharmacy discuss mistakes	116 (55.5%)
	When patient safety issues occur in the pharmacy, staff discuss them	109 (52.4%)
	In this pharmacy we talk about ways to prevent mistakes from happening	120(57.9%)
**Perception of pharmacists on responses to mistakes**		
**Response to mistakes**	Staff are treated fairly when they make mistakes	140 (67.3%)
	This pharmacy helps staff learn from their mistakes rather than punishing them	126 (61.4%)
	We look at staff actions and the way we do things to understand why mistakes happen in this pharmacy	135 (65.9%)
	Staff feel like their mistakes are held against them	88 (42.5%)
**Organization learning-continuous improvement**	When a mistake happens, we try to figure out what problems in the work process led to the mistake	145 (70%)
	When the same mistake keeps happening, we change the way we do things	130 (62.8%)
	Mistakes have led to positive changes in this pharmacy	129 (62%)

Overall, the survey revealed that there were more positive than negative responses to individual survey items, indicating a generally positive perception of patient safety culture in the community pharmacies surveyed.

Our bivariate and multivariate analyses revealed that age, job description, and experience in the field were significant predictors of the overall perception of patient safety. Specifically, being 35 years or older (26.7%), holding a managerial role (22.2%), and having more than 6 years of experience in the field (18.5%) were all positively associated with an overall positive perception of patient safety (as presented in Table 3).

**Table 3:** Personal and job factors affecting the 11 patient safety culture composites in the studied community pharmacies

Factors	Number and % of positive response																								
	Perception about working in this community pharmacy						Practices of community pharmacies on communication and work pace													Perception on responses to mistakes				Overall perception on patient safety	
	Space and environ		Team work		Staff training		Commun. openness			Patient counselling			Staffing work pressure and pace			Commun. across shifts		Commun. of mistakes		Response to mistakes		Organiz. learning			
	*N*	%	*N*	%	*N*	%	*N*		%	*N*		%	*N*		%	*N*	%	*N*	%	*N*	%	*N*	%	*N*	%
**Gender**																									
Male	39	34.2	55	48.2	42	36.8	30		26.3	42		36.8	2		1.8	30	26.3	39	34.2	9	7.9	48	42.1	20	17.5
Female	30	31.9	44	46.8	27	28.7	26		27.7	36		38.3	2		2.1	17	18.1	26	27.7	12	12.8	43	45.7	9	9.6
*P* value	0.73		0.84		0.22		0.83			0.83			0.85			0.16		0.31		0.25		0.60		0.10	
**Age**																									
25 <	6	16.7	10	27.8	5	13.9	6		16.7	8		22.2	0		0	5	13.9	8	22.2	4	11.1	8	22.2	2	5.6
25-<35	38	30.9	58	47.2	44	35.8	29		23.6	47		38.2	3		2.4	27	22	36	29.3	9	7.3	56	45.5	15	12.2
≥ 35	16	53.3	18	60	16	53.3	14		46.7	14		46.7	1		3.3	8	26.7	14	46.7	6	20	15	50	8	26.7
*P* value	0.002		0.01		0.001		0.01			0.04			0.40			0.20		0.04		0.29		0.02		0.01	
**Post-graduate**																									
Yes	25	39.7	34	54	24	38.1	22		34.9	22		34.9	3		4.8	15	23.8	19	30.2	8	12.7	26	41.3	8	12.7
No	45	31.3	65	45.1	46	31.9	36		25	57		39.6	2		1.4	32	22.2	47	32.6	14	9.7	66	45.8	21	14.6
*P* value	0.24		0.24		0.39		0.14			0.53			0.17			0.80		0.73		0.52		0.54		0.72	
**Position**																									
Regular	47	30.5	72	46.8	50	32.5	37	24		58	37.7		3	1.9		35	22.7	46	29.9	16	10.4	69	44.8	17	11
Manager	21	38.9	26	48.1	18	33.3	20	37		21	38.9		2	3.7		12	22.2	20	37	6	11.1	23	42.6	12	22.2
*P* value	0.26		0.86		0.91		0.07			0.87			0.61			0.94		0.33		0.88		0.78		0.04	
< 3	17	29.3	23	39.7	12	20.7	16	27.6		22	37.9		0	0		13	22.4	16	27.6	4	6.9	21	36.2	4	6.9
3- > 6	21	31.3	35	52.2	22	32.8	17	25.4		23	34.3		2	3		11	16.4	18	26.9	9	13.4	30	44.8	9	13.4
≥ 6	30	37	41	50.6	35	43.2	24	29.6		33	40.7		3	3.7		21	25.9	29	35.8	7	8.6	38	46.9	15	18.5
*P* value	0.33		0.24		0.01		0.76			0.69			0.27			0.54		0.27		0.83		0.22		0.05	

**Fig. 1:**
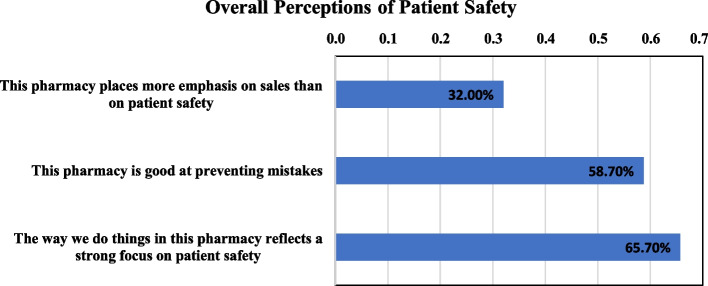
The percentages of positive response for overall perception of patient safety among community pharmacists, Cairo, Egypt, 2019–2020

Figure 1 displays the percentages of positive responses for the overall perception of patient safety. The majority of respondents reported that their pharmacy has good to strong focus on patient safety. However, 32% stated that their pharmacy prioritizes sales over patient safety.

**Fig. 2:**
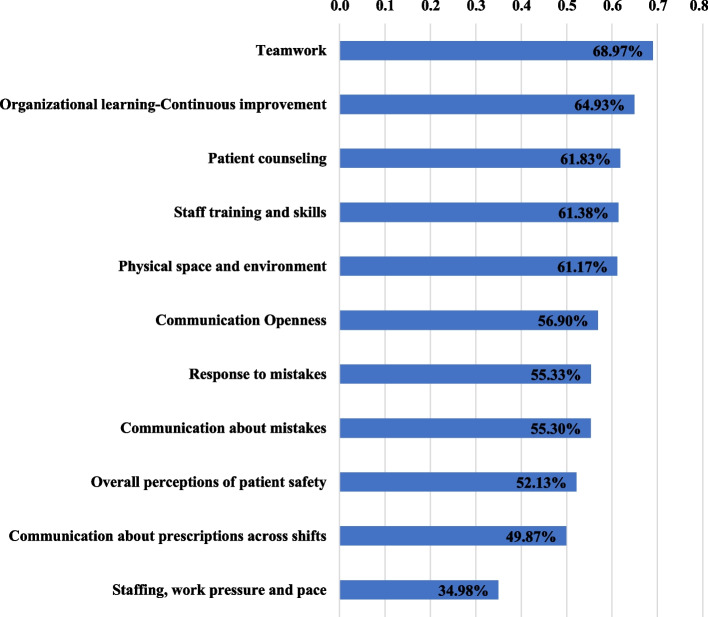
The percentages of positive responses in descending order, for the 11 patient safety culture composites reported by community pharmacy survey among community pharmacists, Cairo, Egypt, 2019–2020

Figure 2 displays the percentages of positive response for the 11 patient safety culture composites in a descending order. The teamwork composite had the highest positive response (69%) while staffing, work pressure and pace composite had the lowest (35%). Except for staffing, work pressure and pace, the average positive response for each composite ranged from 50 to 65%.

## Discussion

The focus of patient safety culture assessments in healthcare has been primarily on hospital settings, with less attention given to community pharmacies. Although patient safety in community pharmacies has been evaluated in several global studies, this study is the first of its kind to be conducted in Cairo to investigate the patient safety culture among pharmacists working in 210 community pharmacies located in the central and southern regions of Cairo.

We found that a considerable proportion of the pharmacies were run without a working pharmacist at the time of the visit. This is an alarming patient safety issue that should be highlighted [9]. The response rate was high (95.5%), which is comparable to a study conducted in Malaysia with a response rate of 93.5% [10], but higher than a study conducted in the Emirates with a response rate of 70.6% [11]. The difference in response rates may be attributed to variations in populations, cultures, and research methods. In this study, the investigator personally visited the community pharmacies, explained the purpose of the study, provided the questionnaires, and returned the following day to collect the completed questionnaires.

The majority of the study participants were junior pharmacists and 26% of them held a managerial role in the pharmacy. These results align with a study conducted in China, where 80% of participants were junior pharmacists. However, the gender distribution differed between the two studies, with women constituting 72% of the Chinese study sample and 54% in this study [12].

The Patient Safety Organizational Patient Safety Culture (PSOPSC) questionnaire measures 11 composites of organizational patient safety culture, six composites measured perception, and five measured practices. The overall mean percentage of positive responses for patient safety culture was 57.4%, which is lower than earlier studies conducted in China, Malaysia, Taiwan, and Kuwait, respectively [7, 10, 12, 13]. This study's findings suggest that patient safety may not be a top priority for community pharmacies in Cairo, despite being a crucial aspect of healthcare quality. This highlights the need for increased focus on patient safety in community pharmacies in the future.

The study also examined pharmacists’ perceptions of their work in community pharmacies across three domains: physical space and environment, teamwork, and staff training and skills. Among these domains, the highest positive response percentage (PRP) was found in the domain of teamwork (68.97%). This result is consistent with previous studies [7, 13, 14] and may be attributed to the small number of personnel and the close proximity of work settings which may facilitate teamwork [15].

The practices of community pharmacies related to communication and work pace were classified into 5 domains. Patient counseling received the highest PRP (61.8%). These results partially agree with a study conducted in Malaysia, where patient counseling PRP was the second highest (78.7%) after the teamwork domain (81%) [10].

These findings are consistent with a study conducted in Qatar, where patient counseling and teamwork composites of patient safety culture had the highest positive responses (95% and 93.7%, respectively), while the “staffing, work pressure, and pace” composite had the lowest positive response (50.6%) among the 11 composites [16].

The results demonstrate the commitment and willingness of pharmacy personnel to spend adequate time with patients. Notably, patient counseling is an essential aspect of pharmacy practice since many community pharmacy service users seek advice from pharmacy personnel during their visits. Comparable results were reported in other studies [7, 12, 16].

In this study, pharmacists’ perceptions of responses to mistakes were classified into two domains: response to mistakes and Organization learning-continuous improvement. The Organization learning-continuous improvement domain received a higher score (64.9%), reflecting pharmacists’ acceptance of a learning culture that promotes patient safety.

This particular area still needs to be enhanced, especially when compared with previous studies on patient safety culture conducted in different demographic regions, where PRP ranged from 81 to 84%. These results were obtained from the 2012 preliminary comparative study, which surveyed 60 pharmacies and 496 staff across the USA, titled “Pharmacy Survey on Patient Safety Culture” [17].

The study also evaluated pharmacists’ overall perception of patient safety through three items: the pharmacy’s focus on sales versus patient safety, the pharmacy’s effectiveness in preventing mistakes, and the pharmacy’s overall commitment to patient safety. Two-thirds of the pharmacists reported that their pharmacies focus more on patient safety, compared to one-third who reported that their pharmacies focus more on sales. Furthermore, 58.7% of pharmacists reported that their pharmacies are good at preventing mistakes, indicating a positive perception of patient safety in community pharmacies.

The results are promising, as the top three PRP of all patient safety composites were for teamwork (68.97%), organization learning-continuous improvement (64.93%), and patient counselling (61.83%). This indicates that practicing pharmacists have a learning culture to improve services that ensure patient safety.

However, the PRP was less than 60% in communication openness (56.90%), response to mistakes (55.33%), communication about mistakes (55.30%), overall perceptions of patient safety (52.13%), and communication about prescriptions across shifts (49.87%). These findings emphasize the importance of improving communication in community pharmacies, which requires collaborative efforts to plan and implement an effective improvement project. Previous studies have highlighted the role of effective communication in reducing problems related to drug prescription and dispensing [18, 19].

The lowest PRP was given to the “staffing, work pressure, and pace domain,” which scored 34.98%. This reflects insufficient staff to handle the workload, which may lead to rushing in handling prescriptions. This finding is consistent with low PRP found in earlier studies conducted in different geographic areas such as Ethiopia (45%) [15] and Malaysia (46.18%) [10]. Moreover, the results of the current study were not far from the results of a cross-sectional survey conducted in a pharmacy department consisting of staff members who provide dispensing, clinical, and support services within an integrated health delivery system in the USA, where PRP of staffing and work pressure was 44.7% [20].

These findings are also consistent with data obtained from three of the largest public hospital pharmacies and three of the largest private hospital pharmacies in Kuwait, where the results of the positive response rate of the 11 composites ranged from 36 to 87%. The lowest score per composite was 36% for “Staffing, work pressure, and pace,” while the highest score was 87% for “Teamwork” [21].

The respondents’ perceptions of inadequacy of staff allocation in handling the overwhelming workload, especially interruptions and distractions in a community pharmacy, emphasize the importance of addressing this issue. This finding is particularly alarming because inadequate staff can severely limit pharmacists’ ability to safely dispense prescriptions, thus increasing the risk of patient harm [2]. Given these drawbacks, it is essential to ensure adequate allocation of pharmacy staff during working shifts and provide all staff with the breaks they are entitled to during each shift. These measures can help reduce medication errors in busy community pharmacies and enhance patient safety culture.

In this study, personal and job-related factors that may affect patient safety culture composites in community pharmacies were also investigated. Age was found to be a significant factor affecting eight patient safety culture composites, while years of experience significantly affected two composites. Pharmacists with managerial roles had a significantly higher overall perception of patient safety. The findings suggest that patient safety culture improves as pharmacists age, which may explain the significant association between years of experience and some patient safety composites. This is consistent with previous studies that also have discussed the influence of age on patient safety culture [22–25]. Moreover, a recent study in Qatar showed that respondents with six or more years of practice experience in significantly responded more positively to survey items, these findings may be related to the fact that greater work experience grants pharmacy personnel the ability to effectively manage patient safety-related issues, as well as adapt to the immense workload adequately, than those with less experience [16].

### Strengths and limitations of the study

This study is novel in addressing how medication safety is handled in community pharmacies in Egypt. The results highlight the need to prioritize patient safety on the top of strategic priorities at the community pharmacy level.

The study was conducted on only 210 pharmacies, representing less than 1% of the total number of community pharmacies in Cairo geographic market, using a convenience sampling method. Therefore, the results may not provide a comprehensive picture of the patient safety culture in all Cairo pharmacies. Additionally, while pharmacists play a crucial role in community pharmacies, it is essential to consider the safety culture of all employees.

## Conclusion

The study found that the overall patient safety culture among community pharmacists is at an average level, with teamwork being a positive aspect being. However, urgent attention is required in areas of weakness, primarily in the domains of “staffing and work pressure,” “response to mistakes,” and “communication of mistakes.” Addressing these issues would necessitate training pharmacists, particularly junior ones, on the principles and significance of patient safety. These training programs could include communication skills, teamwork, and error reporting and documentation. Also, it is important to recognize the role of managerial pharmacists in promoting patient safety culture, and to provide them with the necessary training and resources to effectively lead and manage their teams. Furthermore, the study highlights the universal issue of overwhelming workload in community pharmacies, which requires a collaborative effort to explore and identify viable solutions that are suitable for different cultures and economic statuses.

Additionally, the concept of reporting and documenting medication errors needs to be explored among community pharmacists, and significant effort should be made to promote a culture of reporting and documentation. Conducting a national community pharmacy survey on patient safety culture is highly recommended in Egypt.

## Data Availability

The data set produced during the current study is available on reasonable request.

## Abbreviations

PSOPSC: Pharmacy Survey on Patient Safety Culture
AHRQ: Agency for Healthcare Research and Quality
PRP: Positive Response Percent
USA: United States of America
